# Cardiac Autonomic Modulation in Subjects with Amyotrophic Lateral Sclerosis (ALS) during an Upper Limb Virtual Reality Task: A Prospective Control Trial

**DOI:** 10.1155/2022/4439681

**Published:** 2022-02-09

**Authors:** Ana C. Silveira, Íbis A. P. Moraes, Giovanna P. Vidigal, Amanda O. Simcsik, Renata M. Rosa, Francis M. Favero, Susi M. S. Fernandes, David M. Garner, Luciano V. Araújo, Marcelo Massa, Luiz C. M. Vanderlei, Talita D. Silva, Carlos B. M. Monteiro

**Affiliations:** ^1^Postgraduate Program in Physical Activity Sciences, School of Arts, Science and Humanities of University of São Paulo (EACH-USP), São Paulo, SP 03828-000, Brazil; ^2^Postgraduate Program in Rehabilitation Sciences, Faculty of Medicine, University of São Paulo (FMUSP), São Paulo, SP 01246-903, Brazil; ^3^Department of Neurology and Neurosurgery at Paulista School of Medicine, Federal University of São Paulo (EPM/UNIFESP), São Paulo, SP 04021-001, Brazil; ^4^Department of Physiotherapy, Mackenzie Presbyterian University, São Paulo, SP 01302-907, Brazil; ^5^Cardiorespiratory Research Group, Department of Biological and Medical Sciences, Faculty of Health and Life Sciences, Oxford Brookes University, Headington Campus, Gipsy Lane, Oxford OX3 0BP, UK; ^6^Postgraduate Program in Information Systems, School of Arts, Science and Humanities of University of São Paulo (EACH-USP), São Paulo, SP 03828-000, Brazil; ^7^Department of Physiotherapy, Paulista State University (UNESP), Presidente Prudente, São Paulo, SP 19060-900, Brazil; ^8^Postgraduate Program in Medicine (Cardiology) at Escola Paulista de Medicina, Federal University of São Paulo (EPM/UNIFESP), São Paulo, SP 04021-001, Brazil; ^9^Faculty of Medicine of the City of São Paulo University (UNICID), São Paulo, SP 03071-000, Brazil

## Abstract

Amyotrophic lateral sclerosis (ALS) is a neurodegenerative disease. As a result of the rapid progression and severity of the disease, people with ALS experience loss of functionality and independence. Furthermore, it has already been described presence of autonomic dysfunction. Despite the increasing use of virtual reality (VR) in the treatment of different diseases, the use of virtual reality environment as an intervention program for ALS patients is innovative. The benefits and limitations have not yet been proven. Our objective was to evaluate the autonomic function of individuals with amyotrophic lateral sclerosis throughout the virtual reality task. The analysis of autonomic function was completed before, during, and after the virtual reality task using the upper limbs; also, all steps lasted ten minutes in a sitting position. Heart rate variability (HRV) was taken via the Polar® RS800CX cardiofrequencymeter. The following questionnaire was enforced: Amyotrophic Lateral Sclerosis Functional Rating Scale-Revised (ALSFRS) and Fatigue Severity Scale (FSS). Different types of HRV were revealed for the groups, indicating that the ALS group has reduced HRV, with most of the representative indices of the sympathetic nervous system. Besides, the physiological process of reducing parasympathetic activity from rest to VR activity (vagal withdrawal), with reduction in HF (ms^2^) and an increase in HR from rest to activity, and a further increase throughout recovery, with withdrawal of sympathetic nervous system, occurs just for the control group (CG), with no alterations between rest, activity, and recovery in individuals with ALS. We could conclude that patients with ALS have the reduction of HRV with the sympathetic predominance when equated to the healthy CG. Besides that, the ALS individuals have no capability to adapt the autonomic nervous system when likened to the CG during therapy based on VR and their recovery.

## 1. Introduction

Amyotrophic lateral sclerosis (ALS) is a neurodegenerative disease categorized by progressive loss of upper and lower motor neurons [1]. It affects the motor neurons of the cerebral cortex, brainstem, and spinal cord in adults [2–4]. Individuals with ALS live with continuous and multiple functional losses, presenting upper, lower, and trunk muscle atrophy, muscle weakness, fasciculation, and subsequently progressive loss of all movements [5, 6].

Although this population has chiefly motor symptoms, autonomic nervous system (ANS) disorders have also been reported, such as decreased heart rate variability (HRV) [7–10] with chronic cardiac sympathetic hyperactivity, that is, allied in this population with sudden cardiac death and stress-induced cardiomyopathy [11, 12].

As said by Vanderlei et al. [13], the ANS has a key role in regulating the physiological processes of the human organism throughout both normal and disease conditions. HRV has been established as a cheap technique that allows individual analysis of sympathetic and parasympathetic activity in many situations [14].

Deviations in HRV patterns provide a sensitive and early indicator of the physiological behavior of the human organism and the person's health [12]. A high HRV is a sign of good physiological adaptation, with efficient autonomic mechanisms, whereas a low HRV is an indicator of abnormal and insufficient ANS adaptation, both implying the presence of physiological changes in the individual [15].

In view of the above discussion, one possibility to analyze HRV is the use of physical activities where individuals are assessed during rest and movement activities. So, an innovative technology to offer physical activity for individuals with ALS is the use of virtual reality task. According to Dontje et al. [16], professionals involved in the rehabilitation of patients with ALS search for interventional ideas based on scientific evidence [16] and virtual reality has been considered an interesting option.

A bespoke technology, such as virtual reality (VR), defined as the use of interactive simulations, created with computer hardware and software, to present users with opportunities to perform in virtual environments that appear like real-world objects and events, has been promoted as a tool to stimulate movement and to support individuals to gain improvements in physical performance [17].

Few studies with ALS are directed to the computational technology. Chang et al. [18] analyzed three patients with ALS in a program using brain-computer interface (BCI) expertise, which offered the ability to verify accuracy and information transfer rate in the disabled group as compared to the typically developing group. Thompson et al. [19] and Silvoni et al. [20] steered research on BCI in ALS subjects and demonstrated possibilities for communication at advanced stages of the disease. Lancioni et al. [21] substantiated function in tasks with the use of computer mouse for interaction with written texts. Trevizan et al. [22] applied a VR task with alternative computer interfaces in ALS patients and attained some positive results in motor improvement with repetition.

Considering the lack of studies that analyze the ANS disorder in ALS subjects throughout physical activity, this study enforced HRV to investigate acute cardiac autonomic responses during rest, VR tasks, and recovery. Likewise, we organized a control group with a typical individual matched by gender and age with the ALS group that went through the same protocol to deliver a normal HRV pattern. Hence, if the HRV responses of ALS participants during virtual tasks are upgraded compared with the responses at rest, it provides new ways for research using technology that could be therapeutic by refining autonomic dysfunction in this cohort.

## 2. Materials and Methods

This is a prospective controlled trial. In this study, there were 41 age-matched individuals split into groups with diagnosis of ALS and healthy typically developed as a control group (CG). All subjects who were diagnosed with ALS were confirmed by medical analysis and regularly attended the Neuromuscular Disease Research Sector (SIDNM) of the Federal University of São Paulo, Brazil.

Subjects were omitted with severe motor problems and those with deviations in cognitive functions that would hinder the basic understanding of commands in the proposed activities. The research project (number CAAE: 80826217.0.0000.5390) was approved by the research ethics committee of the University of São Paulo and commenced after the signing of the Terms of Free and Informed Consent by the participants and was registered in the http://ensaiosclinicos.gov.br database with the subsequent number identifier: RBR-78zmw.

Data collection forms from their medical records were finalized in ALS individuals. It was enforced to acquire relevant information regarding patients' care, such as mass, height, date of birth, disease history, pharmacotherapies, and if they were a smoker or former smoker.

For the participants' clinical features, three scales referring to functional assessment, fatigue, and quality of life were necessary:

1. As a functional assessment tool, the Revised Amyotrophic Lateral Sclerosis Functional Rating Scale (ALSFRS-R) was enforced, validated in Brazilian individuals with ALS which permits monitoring of symptoms and limitations of daily living activities. The scale has 12 questions, with scores ranging between 0 and 4 and a maximum score of 48 (where the participant is in his or her optimum state) [23–26]
2. To assess fatigue during the task implementation, we enforced the Fatigue Severity Scale (FSS). The FSS contains nine declarations, and for each item, subjects are instructed to choose a score ranging from 1 to 7, with 7 representing the highest level of agreement with a given statement. The total FSS score is obtained by calculating the mean of all items, with a score ≥ 4 indicating the presence of fatigue [27, 28]

### 2.1. Data Collection Instruments

#### 2.1.1. MoveHero

**Figure 1:**
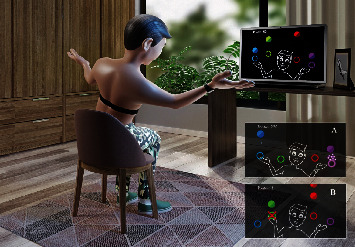
Representative design of the accomplishment of the MoveHero software task in the use of the webcam interface, using the Polar RS800CX chest strap ECG measuring device. (a) Demonstration of hit performed by the participant. (b) Error performed by the participant.

The virtual reality task commenced was a computer game named “MoveHero” that provided an intervention via a virtual task. According to Martins et al. [29] and Silva et al. [30, 31], the game presents tumbling spheres on the computer screen, in four imaginary columns, with a musical rhythm chosen by the researcher. The action is to react (using the upper limbs) and not let the spheres pass from the fixed targets. The spheres ought to be only intercepted when they reach the targets allocated in parallel (at two height levels), two on the left (left position targets A and B) and two on the right (right position targets C and D) of the participant, as illustrated in Figure 1.

The virtual contact was accomplished by the individuals' avatar, namely, a representation of the individual appeared on the computer screen. The individual motivated their upper limbs in front of the webcam to coincide with the moment the ball touched the target. The avatar's hand must reach the target sphere together with the arrival of the ball, and the game proposes feedback on correctness and error by means of changing the spheres' colour (green for correct and a red line for error). The game requires the subject to have a plan for the movement and its anticipation; this is likewise considered a coincident timing task. The subjects were located one meter from the computer monitor and they waited for the spheres to drop, which fell arbitrarily on each target.

#### 2.1.2. Heart Rate Variability (HRV)

We applied HRV to assess the autonomic nervous system before, during, and after the intervention. The data collection strap was situated on the participant's chest, and the HRV was logged using the Polar RS800CX chest strap ECG measuring device (Polar Electro Oy, Kempele, Finland), formerly validated for beat-to-beat heart rate capture, with the heart rate receiver placed next to it.

Prior to HRV measurements, blood pressure, heart rate, and general state of the individual were measured, as measures regarding their activity. HRV was logged after the initial assessments, subjects persisted at rest in the sitting position for 20 minutes, while practicing “MoveHero” for 10 minutes, and immediately after the practice for 10 minutes, we considered this the recovery period.

For assessment, HRV was logged for no less than 10 minutes, to assess at least 256 consecutive RR intervals (after filtering). Moderate digital filtering was completed in the program itself, to eliminate premature ectopic beats and artefacts [13, 32], and there was no substitution, only the elimination of artefacts. Only series within excess of 95% sinus beats were included in the study [33].

HRV analysis was executed using linear (time and frequency domain) and nonlinear methods, studied via Kubios HRV® software (Kubios HRV v.1.1 for Windows, Biomedical Signal Analysis Group, Department of Applied Physics, University of Kuopio, Finland).

#### 2.1.3. HRV Indices

Time-domain analysis was achieved by RMSSD, which is the square root of the mean of squared differences between successive beat intervals, and PNN50 that corresponds to the percentage of adjacent RR intervals that differ by more than 50 milliseconds (ms), SDNN that corresponds to the standard deviation of all normal RR intervals, and similarly mean RR and mean HR [13, 34–36].

For the frequency domain, the spectral components of low frequency (LF: 0.04 Hz to 0.15 Hz) and high frequency (HF: 0.15 Hz to 0.40 Hz) were estimated, in milliseconds squared (ms^2^) and normalized units (n.u.), LF/HF ratio. The spectral analysis was calculated via the fast Fourier transform (FFT) algorithm [37].

A Poincaré plot translates RR intervals into geometric patterns and permits HRV analysis via the geometric or graphic properties of the resultant patterns [38]. Thus, SD1 is the short-term variability of continuous RR intervals, SD2 is the long-term variability of continuous RR intervals, and then there is the SD1/SD2 ratio. The Poincaré plot analysis is considered based on nonlinear dynamics [39].

### 2.2. Collection Procedures

**Figure 2:**
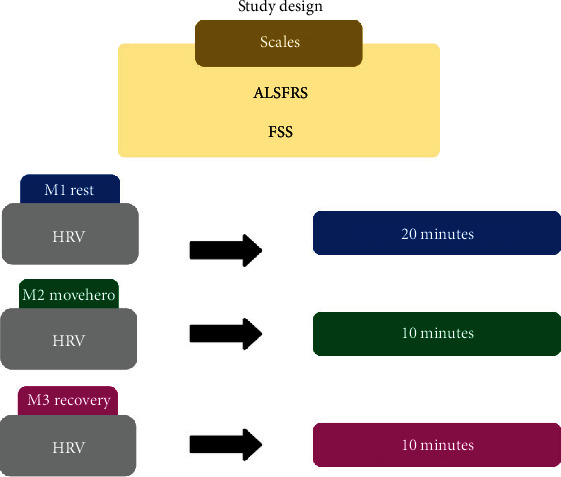
Flowchart of experimental design. ALSFRS: Amyotrophic Lateral Sclerosis Functional Rating Scale-Revised; FSS: Fatigue Severity Scale; HRV: heart rate variability; M1: first moment of HRV evaluation; M2: second moment of HRV evaluation; M3: third moment of HRV evaluation.

Amyotrophic Lateral Sclerosis Functional Rating Scale-Revised (ALSFRS) and Fatigue Severity Scale (FSS) were initial assessments to characterize the participants. The HRV receptor was situated on the patients' wrist and strap placement on their chest; the individuals from both groups remained at rest and were seated in a chair with spontaneous breathing for 20 minutes. After this period, the computer screen was initiated, and the persons remained seated with a laptop computer to enable them to perform the game on the computer for 10 minutes. HRV was evaluated throughout three time periods: the period before (M1—rest), during (M2—“MoveHero” activity), and for ten minutes following the motor task (M3—recovery) (Figure 2). The participants were appraised individually in a suitable room with a laptop computer, desk, and chair (Figure 1) and evaluator involvement responsible for instruction and the data collection annotation.

### 2.3. Statistical Analysis

Descriptive statistics were completed. Categorical data was expressed as absolute and relative frequencies while continuous data were expressed as average ($\bar{x}$) and standard error (se). Data normality was judged by a histogram with normality curve analysis. The following tests were enforced to verify the differences in each HRV index between the groups: multiple analysis of variance (MANOVA, intergroup evaluation) with repeated measures for comparison between the evaluation moments (M1—rest; M2—“MoveHero” activity; M3—recovery; intragroup assessment) with least significant difference (LSD) as a post hoc test. Partial eta-squared (*η*^2^) was conveyed to measure effect sizes and interpreted as small (effect size > 0.01), medium (effect size > 0.06), or large (effect size > 0.14) [40]. Values of *p* < 0.05 (or <5%) were considered significant. The statistical package applied was Statistical Package for the Social Sciences (SPSS; IBM, Chicago, Illinois, USA), version 20.0.

## 3. Results and Discussion

Data collection was carried out from March 2017 to October 2018. A total of 49 potential subjects were requested to participate in the study. Six participants with ALS and 2 participants of the control group were omitted as they did not complete the protocol. Following exclusion criteria, 41 subjects were included in the data analysis, 21 with ALS and 20 from the control group. The groups were homogeneous concerning age, weight, and height. The sample characterization is represented in Table 1.

During HRV analysis, the MANOVA revealed a significant main effect for moments (Wilks' lambda = 0.300, *F*56, 387 = 2.51, *p* < 0.001, *ηp*^2^ = 0.26). No significant main effect for group or interaction between moments and group was found. Certainly, the separate ANOVAs presented a trend of difference in TINN (ms) and LF (ms^2^) and significant effects for mean RR (ms), mean HR (bpm), and SD2 (Table 2).

Also, the post hoc analysis demonstrated some changes that were not found by the ANOVAs, in which the ALS group revealed reductions from rest to “MoveHero” activity on VR in mean RR, RR Tri, LF/HF ratio, and SD1/SD2 ratio and growths in RMSSD, TINN, HF ms^2^, and SD1. From the activity to recuperation, the ALS group subjects had increased mean RR, RR Tri, TINN, and LF/HF ratio, and from rest to recuperation, there were increases in RMSSD, TINN, and SD1. With regard the control group, from rest to activity, only mean RR and RR Tri decreased, and the matching indices increased from activity in VR to recuperation. Effects and measures of central tendency are represented in Table 2.

## 4. Discussion

The current study assessed HRV responses throughout a virtual task in ALS individuals (experimental group) and a control group (individuals presented with typical development) for three moments (rest, during VR activity, and recovery). We will now discuss the results.

### 4.1. HRV at Rest

The current study established that the experimental group at rest demonstrated lower HF n.u. and higher LF n.u. and LF/HF supporting Sachs et al. [41], suggesting larger sympathetic activation than the control group. Similarly, Dalla Vecchia et al. [42] revealed a greater sympathetic modulation at rest, related to a decrease in HF (ms^2^), so ANS in ALS patients was impaired and these individuals had different baseline patterns, which can be elucidated by differences regarding the site of the lesion in the central nervous system (CNS); resultant in different behaviors of the ANS studies establishes that CNS damage in ALS can lead to degeneration of spinal cords' sympathetic ganglia in the more progressive cases [43–45]. Thus, the presence of different autonomic profiles at rest reveals the new notion of ALS as a multisystem disorder with phenotypic heterogeneity.

According to Desport et al. [46], ALS subjects have a larger resting energy expenditure (i.e., energy vital for the maintenance of normal body functions and homeostasis) when likened to a control group, which can be linked with the presence of sympathetic overactivity at rest and can perhaps predict the rate of disease progression. While there is decrease in HRV with the severity of the disease, the subjects in our study were not in advanced disease stages (namely, they were able to move, climb stairs, and others), but nonetheless, there was an alteration compared to the control group.

It is recognized that incomplete autonomic system functionality can initiate a metabolic adjustment in ALS subjects [9]. Dupuis et al. [47] revealed that ALS patients have hypermetabolism, and nutrient ingestion and absorption are impaired [48]. It can be related to a pathophysiological process in the production of mitochondrial energy and in the sympathoadrenergic activation [46]. Additionally, ALS subjects regularly have high levels of plasma norepinephrine and catecholamines [42] and the sympathoadrenergic system can persevere when the heart is under stress [36], which occurs to those subjects even during rest.

### 4.2. HRV during VR Activity

Another significant discovery was during the performance of the virtual task (activity moment); the parasympathetic modulation declined from rest to activity in the control group at various rates (RMSSD, pNN50, HF (ms^2^), HF (n.u.), and SD1), while HR increased (as expected). Conversely, we revealed an increase in HR also in the experimental group, but there was only a reduction in the HF index (ms^2^). This result highlights a parasympathetic nervous system dysfunction, which did not adjust to the demands of motor and/or cognitive activities in ALS individuals, in an identical intensity when equated to the control group. Yet, we can theorize that this dysfunction extends to the sympathetic system, as both need to act together, as revealed in LF n.u. index (that has part of its components representing sympathetic activation) a tendency to increase during the activity in the control group, but not the experimental group.

Consistent with Acharya et al. [49], sympathetic stimulation occurs in response to stress, exercise, and heart disease, triggering an increase in HR by elevating the rate of firing of pacemaker cells in the hearts' sinoatrial node and parasympathetic modulation, consequential on internal organ function, trauma, allergic reactions, and inhalation of irritating substances, decreasing the firing rate of pacemaker cells and HR, providing a regulatory balance in physiological autonomic function.

Therefore, the reduced HRV achieved in ALS subjects during VR task in this study demonstrated a sympathetic predominance in these individuals, which needs further investigation by reason of the cardiovascular neural profile that can initiate poor clinical results [42], for instance, loss of left ventricular dynamics [50], increased cardiac arrhythmias, cardiovascular mortality [10, 51], and the possible risk of sudden death [12, 36].

### 4.3. HRV during Recovery

Regarding the recovery period, in the control group, there was an upsurge in PNS with a reduction in heart rate when associated to the moment of activity, but lacking the expected return to rest values, probably owing to the short rest time (5 min) after activity. Yet, in the ALS group, these ANS adjustments were not observed. We can speculate that the lack of ANS adaptation in ALS individuals during activity did not permit the PNS to reduce physiologically during activity, so the recovery response did not occur [52].

Another important corresponding finding of the current study was that ALS individuals presented several arrhythmia points during the analysis of the tracings. It has been advised that arrhythmias are marker of cardiac sympathetic denervation, usually present in ALS individuals, and it may possibly incline patients to fatal cardiac events. Pimentel et al. [10] suggested that autonomic dysfunction could affect the ALS clinical progression and increase the cardiac arrhythmia risk and sudden death. Otherwise, of the nonlinear HRV assessments, we used SD1 and SD2, but we know that several nonlinear HRV methods have been used and developed; evidence demonstrates a prognostic role of this analysis in identifying individuals at risk of cardiovascular events or mortality [53]. These analyses consider the application of complex system theory to physiology states and are associated with increased complexity variability when compared to healthy controls [54].

### 4.4. Final Comments and Limitations

Our study was a cross-sectional intervention, where a VR task was necessary to provide motor and/or cognitive activity; conceivably, for this short-term intervention the main results arose in the control group only; however, a limitation of our study is that although most participants from both groups (control and ALS) had completed higher education, without statistical difference between them, we were not able to obtain data on the participants' cognitive status; these data could have generated important responses to our study. A recent meta-analysis of Park et al. [55] demonstrates that therapeutic exercise seems beneficial for ALS patients, in longitudinal studies. While our results did not reveal any physiological adaptations of the ANS, VR tasks with demand for movement of the upper limbs and subjects with ALS similarly offered increased sympathetic activity at rest. We highlight that VR activities can be enforced to judge the ANS adaptation to characterize ALS patients and trace the prognosis and better clinical treatment and recommended in future studies. Moreover, as an alternative to physical activity, VR interpositions promote a more engaging and motivating modality, improving performance, wellbeing, and the recognized benefits of physical activity [22]. As such, VR should be investigated as a chance to heighten interactions and participation, similarly providing the medical clinicians and professionals the ability to modify the difficulty and repetitions of the tasks, and thereby stipulate interaction along with the current and future physical requirements of the ALS patients.

It is important to mention that, although HRV is among the most sensitive and specific approaches currently available to evaluate cardiovascular autonomic neuropathy, its association with other forms of evaluation would be interesting since the interaction between the regulatory processes of the heart function and peripheral blood flow expands the possibilities of assessing autonomic dysfunction. The literature suggests the evaluation of baroreflex sensitivity, muscle sympathetic nerve activity, plasma catecholamines, and heart sympathetic imaging [56]. Besides that, the assessment by means of photoplethysmography could have been a great association to our analyses, for assessing the underlying status of peripheral blood vessels, and whenever possible, it is suggested using additional physiological cardiovascular measurements, increasing accuracy [57–59]. Additionally, by analysis of the phase coherence between the oscillations in microvascular blood flow and tissue oxygenation, it would be possible to study cardiovascular and microvascular dynamical processes [60]. Considering that in our study the focus was on the adaptation of ANS in people with ALS during physical activity in VR, we did not carry out in association with other assessments, so we suggest that this be done in future studies.

## 5. Conclusions

The results reveal that amyotrophic lateral sclerosis (ALS) subjects have reduced heart rate variability (HRV) with sympathetic predominance when likened to the healthy control group. Also, individuals with ALS do not express adaptive capacity of the autonomic nervous system when equated to the control group throughout virtual reality (VR) activity and recovery.

## Figures and Tables

**Table 1:** Sample characterization.

	ALS (*n* = 21)	CONTROL (*n* = 20)	*p* value
	Mean (standard error) [confidence interval]	Mean (standard error) [confidence interval]	
Age (years)	51.3 (2.8) [31-74]	49.1 (10.7) [6.02-15.38]	0.542
Weight (kg)	73.8 (3.1) [55.5-102]	76.5 (3.9) [45-100]	0.602
Height (cm)	166.1 (2.0) [152–184]	170.8 (2.1) [158–185]	0.126
ALFRS	32.3 (0.9) [23–38]	—	—
FSS	40.4 (3.5) [11-61]	—	—
Symptom's time (months)	44.9 (9.9) [12–204]	—	—

	%(*n*)	%(*n*)	
*Gender*			
Male	71.4 (15)	70 (14)	0.545
Female	28.6 (6)	30 (6)	
*Ex-smoker*	28.6 (6)	0	0.006
Smoker	4.8 (1)	0	0.487
Arterial hypertension	23.8 (5)	0	0.049
*Education level*			
High school	28.6 (6)	50 (10)	0.333
Higher education	66.7 (14)	50 (10)	
Physiotherapy	47.6 (10)	—	—

Data presented as mean (standard error) and 95% confidence interval followed by *p* value of Student's *t*-test for continuous variables and chi-square test with posttest Bonferroni (for comparison between groups) for categorical variables. ALS: amyotrophic lateral sclerosis; CONTROL: control group; kg: kilogram; m: meters; cm: centimeters; ALFRS: Amyotrophic Lateral Sclerosis Functional Rating Scale-Revised; FSS: Fatigue Severity Scale.

**Table 2:** Comparison of HRV indices between moments and groups and ANOVA values for the main effects and interaction.

HRV indices	Groups	Moments						Main effect groups	Main effect moments	Interaction groups × moments
		Rest (M1) Mean (SE) [CI LL; UL]	VR activity (M2) Mean (SE) [CI LL; UL]	*p* (M1 × M2)	Recovery (M3) Mean (SE) [CI LL; UL]	*p* (M2 × M3)	*p* (M1 × M3)	*F*; *p* value; *η*_*p*_^2^ (DF = 1, 26)	*F*; *p* value; *η*_*p*_^2^ (DF = 2, 52)	*F*; *p* value; *η*_*p*_^2^ (DF = 2, 52)
Mean heart rate (bpm)	ALS	82.3 (3.2) [75.6; 88.9]	84.8 (3.4) [77.8; 91.9]	*0.085* ^∗^ ↑	82.5 (3.5) [75.2; 89.8]	*0.041* ↓	—	—	10.0; *<0.001*; 0.28	—
	CONTROL	76.9 (2.9) [70.7; 83.0]	82.1 (2.8) [75.7; 88.8]	*<0.001* ↑	80.1 (3.0) [73.3; 86.9]	*0.038* ↓	*0.019* ↑			
	*p* value	—	—		—					

Mean RR (ms)	ALS	746.4 (28.6) [687.5; 805.2]	724.1 (28.6) [665.3; 782.9]	—	747.5 (31.0) [683.7; 811.2]	*0.026* ↑	—	—	10.9; *<0.001*; 0.30	—
	CONTROL	793.1 (26.6) [738.3; 847.8]	740.9 (26.6) [686.1; 795.6]	*<0.001* ↓	762.0 (28.9) [702.7; 821.4]	*0.030* ↑	*0.007* ↓			
	*p* value	—	—		—					

SDNN (ms)	ALS	21.6 (2.8) [15.9; 27.2]	21.3 (2.4) [16.1; 26.4]	—	23.2 (3.1) [16.7; 29.6]	—	—	—	3.24; *0.050*; 0.11	—
	CONTROL	26.7 (2.5) [21.4; 31.9]	25.3 (2.3) [20.6; 30.0]	—	30.4 (2.9) [24.4; 36.4]	*0.028* ↑	*0.074* ^∗^ ↑			
	*p* value	—	—		—					

RMSSD (ms)	ALS	16.8 (2.2) [12.3; 21.3]	15.4 (1.9) [11.6; 19.3]	—	16.1 (2.3) [11.3; 20.9]	—	—	3.59; *0.069*^∗^; 0.12	2.72; *0.081*^∗^; 0.10	—
	CONTROL	21.6 (2.0) [17.4; 25.8]	19.3 (1.7) [15.7; 22.9]	*0.062* ^∗^ ↓	22.8 (2.2) [18.3; 27.2]	*0.030* ↑	—			
	*p* value	—	—		*0.046*					

pNN50 (%)	ALS	2.82 (1.17) [0.41; 5.22]	1.92 (0.65) [0.59; 3.25]	—	2.26 (0.96) [0.29; 4.23]	—	—	—	2.63; *0.095*^∗^; 0.09	—
	CONTROL	3.81 (1.09) [1.57; 6.06]	2.13 (0.60) [0.89; 3.37]	*0.083* ^∗^ ↓	3.41 (0.89) [1.57; 5.24]	*0.038* ↑	—			
	*p* value	—	—		—					

LF (ms^2^)	ALS	365.7 (84.9) [191.0; 540.4]	325.5 (94.1) [132.1; 518.9]	—	449.2 (126.9) [188.3; 710.1]	—	—	—	—	—
	CONTROL	452.3 (79.1) [289.6; 614.9]	517.6 (87.6) [337.5; 697.7]	—	559.7 (118.2) [316.8; 802.5]	—	—			
	*p* value	*—*	—		—					

HF (ms^2^)	ALS	130.5 (36.6) [55.3; 205.6]	78.8 (22.1) [33.5; 124.2]	*0.059* ^∗^ ↓	123.0 (35.8) [49.4; 196.6]	—	—	—	6.52; *0.004*; 0.20	—
	CONTROL	190.7 (34.0) [120.8; 260.7]	134.5 (20.5) [92.3; 176.6]	*0.029* ↓	196.1 (33.3) [127.6; 264.7]	*0.022* ↑	—			
	*p* value	—	*0.076* ^∗^		—					

LF n.u.	ALS	78.3 (3.6) [71.0; 85.7]	81.1 (3.1) [74.8; 87.5]	—	78.2 (2.7) [72.6; 83.9]	—	—	8.07; *0.009*; 0.24	—	—
	CONTROL	66.3 (3.3) [59.5; 73.2]	74.1 (2.9) [68.2; 80.0]	*0.077* ^∗^ ↑	72.4 (2.6) [67.1; 77.6]		*0.084* ↓			
	*p* value	*0.021*	—		—					

HF n.u.	ALS	21.6 (3.6) [14.3; 28.9]	18.8 (3.1) [12.5; 25.1]	—	21.7 (2.7) [16.1; 27.4]	—	—	8.04; *0.009*; 0.24	—	—
	CONTROL	33.6 (3.3) [26.8; 40.4]	25.7 (2.9) [19.8; 31.6]	*0.072* ^∗^ ↓	27.6 (2.6) [22.3; 32.8]	—	*0.083* ^∗^ ↑			
	*p* value	*0.020*	—		—					

LF/HF	ALS	4.55 (0.63) [3.26; 5.84]	5.34 (0.77) [3.74; 6.93]	—	5.35 (0.88) [3.54; 7.16]	—	—	4.77; *0.038*; 0.16	—	—
	CONTROL	2.68 (0.59) [1.48; 3.88]	4.03 (0.72) [2.55; 5.51]	—	3.11 (0.82) [1.42; 4.79]	—	—			
	*p* value	*0.038*	—		*0.073* ^∗^					

SD1 (ms)	ALS	11.9 (1.6) [8.7; 15.1]	10.9 (1.3) [8.2; 13.7]	—	11.4 (1.6) [8.0; 14.8]	—	—	3.60; *0.069*^∗^; 0.12	2.75; *0.079*^∗^; 0.10	—
	CONTROL	15.3 (1.4) [12.3; 18.3]	13.7 (1.2) [11.1; 16.2]	*0.060* ^∗^ ↓	16.1 (1.5) [12.9; 19.3]	*0.029* ↑	—			
	*p* value	—	—		*0.045*					

SD2 (ms)	ALS	27.9 (3.6) [20.5; 35.4]	27.9 (3.4) [20.9; 34.8]	—	30.6 (4.2) [22.0; 39.1]	—	—	—	3.26; *0.050*; 0.11	—
	CONTROL	34.3 (3.4) [27.4; 41.3]	32.9 (3.1) [26.5; 39.4]	—	39.7 (3.9) [31.8; 47.7]	*0.034* ↑	*0.063* ↓			
	*p* value	—	—		—					

SD1/SD2	ALS	2.51 (0.16) [2.19; 2.84]	2.83 (0.22) [2.39; 3.28]	—	2.88 (0.19) [2.50; 3.27]	—	*0.020* ↑	—	3.18; *0.052*; 0.11	—
	CONTROL	2.27 (0.15) [1.97; 2.58]	2.42 (0.20) [2.01; 2.84]	—	2.48 (0.17) [2.12; 2.83]	—	—			
	*p* value	—	—		—					

Data presented as mean (standard error (SE)) and 95% confidence interval (CI) presented in lower limit (LL) and upper limit (UL) followed by the *p* value of the post hoc LSD (least significance difference) test. Post hoc tests were calculated even when there were no significant interactions to find significant results; ^∗^marginally significant results (*p* = 0.055–0.095); arrows indicate whether the values represent an increase (↑) or decrease (↓) of the indices between moments. HRV: heart rate variability; M1–M3: moments 1 to 3; VR: virtual reality (during virtual task); bpm: beats per minute; HF: high frequency; LF: low frequency; LF/HF: low frequency and high frequency ratio; ms: millisecond; ms^2^: millisecond squared; n.u.: normalized units; RMSSD: square root of the mean squared differences of successive RR intervals; RR intervals: intervals between heartbeats; SDNN: standard deviation of the mean of all RR intervals over a period; pNN50: percentage of adjacent RR intervals with a difference in duration greater than 50 milliseconds; SD1: the standard deviation of the instantaneous variability of the beat-to-beat RR interval in ms; SD2: the standard deviation of the long-term continuous variability of the RR interval in ms; SD1/SD2: ratio of short and long variations of RR intervals; ALS: amyotrophic lateral sclerosis group; CONTROL: control group.

## Data Availability

The heart rate variability data used to support the findings of this study are included within the article.
